# Variant formation and distribution of the superficial palmar arch

**DOI:** 10.4103/0970-0358.63942

**Published:** 2010

**Authors:** Srinivasa Rao, Venkata Ramana Vollala, Narendra Pamidi, Somayaji Nagabhooshana, Bhagath Kumar Potu

**Affiliations:** Department of Anatomy, Manipal University, Manipal, India

**Figure 1:**
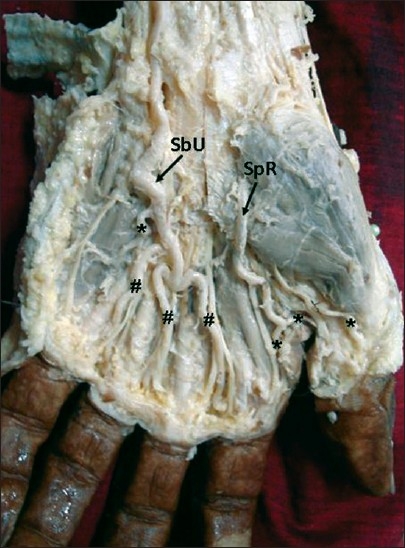
Dissection of the left palm showing the superficial palmar arch formed solely by the superficial branch of the ulnar artery. #Common palmar digital arteries; *Proper palmar digital branches; SbU, superficial branch of the ulnar artery; SpR, Superficial palmar branch of the radial artery

Sir,

The anastomoses between radial and ulnar arteries in the palm play a significant role in diseases of the palm through collateral circulation. During routine dissection of the left upper limb of a 45-year-old male cadaver, we observed the superficial palmar arch (SPA) formed exclusively by the superficial branch of the ulnar artery [Figure 1]. The superficial palmar branch of the radial artery entered the hand through the thenar muscles and provided palmar digital branches to the radial side of the index finger and the ulnar side of the thumb, without any contribution to the SPA. However, the radial side of the thumb was supplied by a branch from the deep palmar arch. The superficial branch of the ulnar artery gave origin to three common palmar digital arteries to supply the contiguous sides of the index, middle, ring and little fingers. It also provided origin to a digital branch to the ulnar side of the little finger.

The superficial arteries of the hand formed several diversified patterns that permitted into well-defined categories. About one-third of the SPA is formed by the ulnar artery alone; a further third is completed by the superficial palmar branch of the radial artery and a third either by the arteria radialis indicis or by the princeps pollicis or by the median artery.[1] A classic type of SPA in which the superficial branch of the radial artery joins the superficial branch of the ulnar artery is found only in 34.5% of the cases.[1,2] There are many reports regarding formation of SPA. In a study by Coleman *et al*., the complete arch was found in 78.5% of the cases and incomplete arch in the remaining 21.5%, and this formed a major underlying factor in the aetiology of digital ischaemia.[3] Ikeda *et al*. conducted stereoscopic arteriography of 220 cadaver hands and reported complete SPA in 96.4% of the cases, and only 3.6% had an incomplete arch.[4] Gellman *et al*. showed a complete SPA in 84.4%[5] and Al Turk and Metcalf reported complete SPA in 84% of the cases.[6] Knowledge of the anatomical variations of the arterial pattern of the hand is crucial for safe and successful hand surgery. The hand surgeon should keep in mind this kind of variation while performing surgical procedures such as, arterial repairs, vascular graft applications and free and/or pedicled flaps. While harvesting the radial artery for use as arterial by-pass conduits or while harvesting the free Radial Forearm Flap, the need to look specifically for variation in collateral circulation, like presence of incomplete SPA[7]. is a must. Currently, the methods of assessing hand circulation include the modified Allen test, Doppler ultrasonography and photoplethysmography. Doppler study is a useful tool in pre-operative screening for radial artery harvesting for myocardial revascularisation.[8]

SPA is an anastomosis, fed mainly by the ulnar artery. When the ulnar artery is occluded, the viability of the structures in the palm supplied by the ulnar artery depends on the efficacy of the collateral circulation. In the present case, there was no anastomosis between the ulnar artery and the radial or median or interosseous arteries. Thus, in ulnar artery occlusion in cases like ours, there will be no collateral flow of blood to meet the metabolic demands of the palmar tissue, and this will result in acute ischaemia, manifested by claudications, rest pain and/or gangrene.
